# Water Safety in California Public Schools Following Implementation of School Drinking Water Policies

**DOI:** 10.5888/pcd17.200366

**Published:** 2020-12-31

**Authors:** Isioma L. Umunna, Lauren S. Blacker, Christina E. Hecht, Marc A. Edwards, Emily A. Altman, Anisha I. Patel

**Affiliations:** 1 Stanford University, Stanford, California; 2 Nutrition Policy Institute, University of California, Oakland, California; 3 Virginia Tech, Blacksburg, Virginia; 4 University of California, Berkeley School of Public Health, Berkeley, California

## Abstract

**Introduction:**

Recent legislation requires public and charter schools in California to test drinking water for lead. Our objective was to describe 1) results from this testing program in the context of other available water safety data and 2) factors related to schools and water utilities associated with access to safe drinking water in schools.

**Methods:**

Our study focused on a random sample of 240 California public and charter schools. We used multivariable logistic regression, accounting for clustering of tested water sources in schools, to examine school-level factors associated with failure to meet lead-testing deadlines and any history of water utility noncompliance.

**Results:**

Of the 240 schools, the majority (n = 174) tested drinking water for lead. Of the schools tested, 3% (n = 6) had at least 1 sample that exceeded 15 parts per billion (ppb) (California action level) and 16% (n = 28) exceeded 5 ppb (bottled water standard). Suburban schools had lower odds of being served by noncompliant water systems (OR = 0.17; CI, 0.05–0.64; *P* = .009) than city schools. Compared with city schools, rural schools had the highest odds of not participating in the water testing program for lead (OR = 3.43; CI, 1.46–8.05; *P* = .005). Hallways and common spaces and food services areas were the most frequent school locations tested; one-third of all locations sampled could not be identified.

**Conclusion:**

In our study, geography influenced access to safe drinking water in schools, including both water utility safety standards and school lead-testing practices. Considerations for improving the implementation of state lead-testing programs include establishing priority locations for sampling, precisely labeling samples, and developing well-defined testing and reporting protocols.

**Summary**

**What is already known on this topic?**

Consumption of lead or other contaminants from drinking water is responsible for several health issues during childhood development. Some states have launched testing programs in schools for lead in drinking water, but information on these programs is lacking.

**What is added by this report?**

Ours is the first postimplementation examination of overall water quality in schools that includes both data on health standards compliance for water systems and results of tests for lead in drinking water.

**What are the implications for public health practice?**

Findings regarding facilitators and barriers to successful water testing in schools can be used to improve the development and implementation of water safety policies in the United States and elsewhere.

## Introduction

Consumption of safe drinking water is essential to health (1); however, concern that available water is unsafe discourages consumption (2). Universal access to safe drinking water remains an unmet goal. Ingesting contaminated water can negatively affect health by increasing cancer risk and impairing cognitive development (3). Because most deleterious effects of contaminated water result from long-term exposure (3), ensuring that children have access to safe water is critical. Children spend substantial time at school, making schools important intervention sites (4).

Although water utilities are charged with ensuring that drinking water meets federal and state water quality standards, lead may still enter school drinking water on the way from the water supplier from lead-containing service lines, pipes, drinking water fixtures, or solder. Only a few studies have focused on the accessibility of safe drinking water in schools (5–7). Some states have launched programs that require schools to test for lead (8), the most common drinking water contaminant. However, testing protocols, funding, and oversight responsibility are inconsistent, as are the remediation methods used (8).

Although evidence supports the importance of lead-testing initiatives in schools (9,10), ours is one of the first studies to describe the passage of legislation requiring lead testing in schools (11) and implementation of California’s mandatory school lead-testing program following the testing deadline specified in the legislation. In our study, we describe the implementation of the program, drinking water system violations, and school-level factors that influence overall school water quality. Evidence suggests that water system violations and limited access to safe drinking water disproportionately affect low-income, racial/ethnic minority populations (9,12). We hypothesized that schools serving these populations would be more likely to have water quality violations, including exceeding recommended lead levels, and would be less likely to comply with the state-mandated testing program.

## Methods

### Setting and participants

We used data from a 2016–2018 cross-sectional parent study that our team completed of access to drinking water in schools and quality practices and policies (6,7). We conducted telephone interviews with school principals or other school staff members who were knowledgeable about drinking water access and quality-related policies and practices. Our study consisted of 240 California public and charter schools, stratified by school type and urban-centric geography, and randomly selected from 10,481 eligible schools. We excluded schools with a nonstandard grade configuration, such as kindergarten to 12th grade, special education schools, vocational schools, and alternative schools (6,13). Details about the parent study are described elsewhere (6,7). For our study, we linked the parent study data set to water quality data on water system compliance and lead-testing participation collected from the California State Water Resources Control Board (SWRCB) from January through October 2019.

Adjusting for the total number of public schools in California, an estimated sample design effect of 1.3, and an anticipated response rate of 80%, we estimated that a sample size of 240 schools achieved >80% power at the 5% significance level to detect an absolute difference in proportions of 15% for likely moderate-prevalence outcomes and 10% for likely low-prevalence outcomes between 2 groups for the parent study. This study was deemed exempt by Stanford University’s institutional review board.

### Study outcomes

**Drinking water system noncompliance.** The first outcome examined was whether a school had a history of water system noncompliance or violation of a water system standard. Compliance data were sourced for each school from SWRCB’s Exceedance/Compliance Status of Public Water Systems database (14). Each water system has a public water system identification number, a unique alphanumeric indicator assigned to all water systems in the state. “Out of compliance” indicated that the water system was in violation of a health and safety regulation at the time of analysis. “Returned to compliance” indicated the water system had violated a safety regulation between 2013 and 2019 but had addressed the violation, returning to compliant status. “In compliance” referred to water systems that had had no health or safety standards violations since January 2013. All water quality violations were health standard violations, not procedural violations.

Water systems identified as out of compliance or recently returned to compliance were categorized as having any history of noncompliance. For schools served by noncompliant water systems and systems recently returned to compliance, we documented data on the dates of noncompliance, the date of return to compliance, the contaminant level for each violation, and the maximum contaminant level allowable.

**Nonparticipation in the testing program for lead in school drinking water**. Our second study outcome was nonparticipation in the mandatory testing program for lead in school drinking water. On October 13, 2017, California passed Assembly Bill 746 (AB 746), mandating lead testing of water supplies in public schools that were built before January 2010 and that used a community water system. This bill required water suppliers to work with public schools to test for lead in the potable water system at the school site before January 1, 2019 (15). AB 746 set 15 ppb as the action level (ie, the level of concentration of a harmful substance that, when exceeded, is considered sufficient to warrant regulatory or remedial action) for lead in drinking water (15).

Nonparticipation in the testing program was defined as not reporting lead-testing results by October 15, 2019. Schools were required to test by July 1, 2019, with a 10-day window for reporting results after testing (15). The October 15 deadline was set to capture data on schools that tested, even if they failed to submit results by the deadline. Data regarding implementation of the school lead-testing program were obtained from the SWRCB database reporting on lead sampling of drinking water in California schools (16). Data captured included schools’ public water system identification number, school testing status, testing date, the location where each drinking water sample was obtained (eg, classroom, bathroom), the type of water source that was tested (eg, fountain, sink), the lead level for each sample, and whether the level exceeded the state action level (15 ppb) (17). Lead levels below 5 ppb were recorded by the state as undetectable and were coded as below the Food and Drug Administration (FDA) action level for bottled water (17).

### Covariates

Covariates were school and student demographic characteristics obtained from the California Department of Education DataQuest database (18). Specific covariates included 1) percentage of students eligible for free and reduced-price meals, which is a proxy for household income; 2) percentage of students from racial/ethnic minority backgrounds (Latino, African American, Asian, American Indian, Pacific Islander, or multiracial); 3) geographic setting of the school identified as city, suburb, rural, or town; and 4) number of students enrolled in the school.

**Data entry, cleaning, and statistical analysis. **Data were entered into Research Electronic Data Capture (REDCap). All entries were checked for errors, verified, and then analyzed by using R, version 3.6.1 (The R Foundation). Descriptive analyses (eg, frequencies, percentages, means) were used to summarize main outcomes and covariates. We used χ^2^ and Fisher exact tests and *t* tests for bivariate analyses of categorical and continuous outcomes. We included covariates associated with outcomes with a significance threshold of *P* ≤.10 in regression analyses. Multivariable logistic regression, accounting for clustering of water sources tested in schools, was used to examine school-level and student-level factors associated with water system noncompliance and nonparticipation in the California lead-testing program. A 2-sided *P* value of <.05 was considered significant, and 95% CIs were calculated for odds ratios (ORs).

Schools with missing data entries were excluded from bivariate tests and logistic regression analyses where that category was compared. No more than 4.2% of the data were missing for any variable. Although there is no established cut-off for an acceptable percentage of missing data for valid statistical inferences, literature suggests that if less than 5% to 10% of the data set is missing, unbiased and valid statistical inferences are achievable (19).

## Results

**Study sample characteristics.** Study schools had a larger student enrollment and a greater proportion of students identified as White than California schools overall (Table 1). The proportion of students who received free and reduced-price meals or who were identified as Latino or African American was not significantly different between study schools and California schools overall.

**Table 1:** Baseline Characteristics of Study Schools (N = 240) and California Schools (10,481)^a^, 2019

Characteristic	Study Schools	California Schools	*P* Value^b^
**Enrollment size, mean (SD)**	798.5 (672.0)	613.9 (536.9)	<.001
**Students eligible for free or reduced-price meals, %**	62.6	62.4	.90
**Race/ethnicity of students, %^b^**			
Latino	52.0	54.0	.27
African American	5.7	5.8	.95
Other race/multiracial	11.7	14.6	.006
White	30.5	24.6	.001

a The 10,481 eligible schools were California public and charter schools, stratified by school type and urban-centric geography. We excluded schools with a nonstandard grade configuration, such as kindergarten to 12th grade, special education schools, vocational schools, and alternative schools (6,13). Percentages do not total 100 because some families did not report race/ethnicity.

b *P* values calculated by using *t* tests to examine differences in school demographic characteristics between study schools and California schools overall.

**Drinking water system noncompliance**. Sixteen percent of schools received water from a water system with a history of noncompliance with water and sanitation regulations, such as elevated levels of contaminants or failure to adhere to disinfectant protocols. In bivariate analysis (Table 2), schools served by water systems with a history of noncompliance were more likely to have a smaller enrollment, be located in a city, and serve >50% of students who were from racial/ethnic minority backgrounds or eligible for free and reduced-price meals. In the adjusted multivariable regression, schools serving predominately minority students were more likely to be served by water systems with a history of noncompliance than schools with fewer students from minority racial/ethnic groups (OR = 3.27; CI, 1.00–10.66; *P* = .05). Additionally, suburban schools had nearly 6 times lower odds of being served by water systems with a history of noncompliance than city schools (OR = 0.17; CI, 0.05–0.64; *P* = .009).

**Table 2:** Student and School Characteristics Associated with Drinking Water System Noncompliance in Study Schools (N = 240), California, 2019

Characteristic	Water System Compliant With Health Standards (N = 195)	Water System With History of Noncompliance With Health Standards (N = 39)	*P* Value for Bivariate Analysis^a^	Odds Ratio of Water System Noncompliance With Health Standards^b^ (N = 228), OR (95% CI)	*P *Value for OR^c^
**Student enrollment, mean**	844.2	650.7	<.001	0.99 (0.99–1.00)^d^	.08
**Geographic setting, n (%)**					
City (reference)	44 (22.6)	17 (43.6)	.02	1.00	Reference
Rural	48 (24.6)	7 (18.0)		0.44 (0.14–1.38)	.16
Suburban	55 (28.2)	4 (10.3)		0.17 (0.05–0.64)	.009
Town	48 (24.6)	11 (28.2)		0.72 (0.28–1.84)	.49
**Students eligible for free or reduced-price meals, n (%)**					
≤50% (reference)	65 (33.3)	5 (12.8)	.01	1.00	.37
>50%	130 (66.7)	34 (87.2)		1.65 (0.55–4.96)	
**Students from racial/ethnic minority^e^ backgrounds, n (%)**					
≤50% (reference)	68 (34.8)	5 (12.8)	.007	1.00	
>50%	127 (65.1)	34 (87.2)		3.27 (1.00–10.66)	.05
**School type, n (%)**					
Elementary	63 (32.2)	13 (33.3)	.93	NS in bivariate	
Middle	66 (33.9)	12 (30.8)			
High	66 (33.9)	14 (35.9)			

Abbreviations: NS, not significant; OR, odds ratio.

a *P *values for bivariate analysis were calculated by using *t* test for continuous data and χ^2^ test or Fisher exact test (in the case of sparse data) for categorical data.

b Covariates with *P* ≤ .10 in bivariate analysis were included in regression analyses. Sample size for the multivariable logistic regression does not equal N=234 because of missing school demographic characteristics.

c *P *value for odds ratios were calculated by using multivariable logistic regression, accounting for clustering of tested water sources in schools.

d Actual values recorded: 0.9993246 (0.9985735, 1.000076).

e Minority racial/ethnic groups are Latino, African American, Asian, American Indian, Pacific Islander, and multiracial.

**Implementation of the California school testing program for lead in drinking water**. Most study schools (n = 174) tested drinking water for lead through the state testing program (Table 3). Of the 66 schools that did not test drinking water for lead through the state program, 54 received water from a community water system. Twelve schools, all of which were from rural and town areas, received water from wells (n = 6) or another unknown water source (n = 6). In bivariate analysis, schools that failed to test taps for lead had fewer enrolled students and were more likely to be located in a rural area than schools that tested. In adjusted analysis, rural and town schools had higher odds of not testing taps by the deadline (rural: OR = 3.43; CI, 1.46–8.05; *P* = .005; town: OR = 2.40; CI, 1.00–5.76; *P* = .05) as compared with city schools.

**Table 3:** Student and School Characteristics Associated with Participation in the California Testing Program for Lead in School Drinking Water, 2019

Characteristics	Water Tested for Lead, N = 174	Water Not Tested for Lead, N = 66	*P* Value for Bivariate Analysis^a^	Odds Ratio of Water Not Tested for Lead^b^, N = 234, OR (95% CI)	*P* Value for OR^c^
**Student enrollment, mean**	862.1	634.2	<.001	0.99 (0.99–1.00)^d^	.28
**Geographic setting, n (%)**					
City (reference)	51 (29.3)	10 (15.2)	.001	1.00	Reference
Rural	34 (19.5)	26 (39.4)		3.43 (1.46–8.05)	.005
Suburban	49 (28.2)	10 (15.2)		1.09 (0.41–2.93)	.86
Town	40 (23.0)	20 (30.3)		2.40 (1.00–5.76)	.05
**Students eligible for free or reduced-price meals, n (%)**					
≤50% (reference)	53 (30.5)	18 (27.3)	.63	NS in bivariate	
>50%	121 (69.5)	48 (72.7)			
**Students from racial/ethnic minority^e^ backgrounds, n (%)**					
≤50% (reference)	53 (30.5)	21 (31.8)	.83	NS in bivariate	
>50%	121 (69.5)	45 (68.2)			
**School type, n (%)**					
Elementary	62 (35.6)	18 (27.3)	.47	NS in bivariate	
Middle	55 (31.6)	24 (36.4)			
High	57 (32.8)	24 (36.4)			

Abbreviations: NS, not significant; OR, odds ratio.

a *P* values for bivariate analysis were calculated by using *t* test for continuous data and χ^2^ test or Fisher exact test (in the case of sparse data) for categorical data.

b Covariates with *P* ≤ .10 in bivariate analysis were included in regression analyses. Sample size for the multivariable logistic regression does not equal N=240 because of missing school demographic characteristics.

c *P *value for odds ratios were calculated using multivariable logistic regression, accounting for clustering of tested water sources in schools.

d Actual values recorded: 0.9996147 (0.999016, 1.000214).

e Minority racial/ethnic groups include Latino, African American, Asian, American Indian, Pacific Islander, and multiracial.

**Results from the California school testing program for lead in drinking water**. Three percent (n = 6) of the 174 schools that tested their taps for lead through the state program had at least 1 drinking water sample that exceeded 15 ppb, the California state action level for lead. Sixteen percent (n = 28) of schools that tested through the program had at least 1 drinking water sample that exceeded 5 ppb, the FDA threshold for bottled water. Of these, 32% (n = 9) were served by water systems that were in violation of water safety standards (none in violation for lead level exceedance). The other 68% of schools with elevated lead levels (n = 19) were served water by utilities in compliance with health and safety regulations.

The 174 schools that tested their taps for lead collectively tested 1,128 independent water sources, averaging 6 sites per school. One-third of all water sources tested were unidentifiable, with nondescript labels such as “Building A” or “Block 3.” The most common identifiable water source was located in hallways and other student common areas (23%), followed by food service areas (13%), and physical activity spaces (12%) such as gyms, and playgrounds. Approximately 8% of all taps tested were in locations such as staff lounges, nurses’ stations, distribution sources, and maintenance areas that were not readily accessible to students (Figure).

**Figure:**
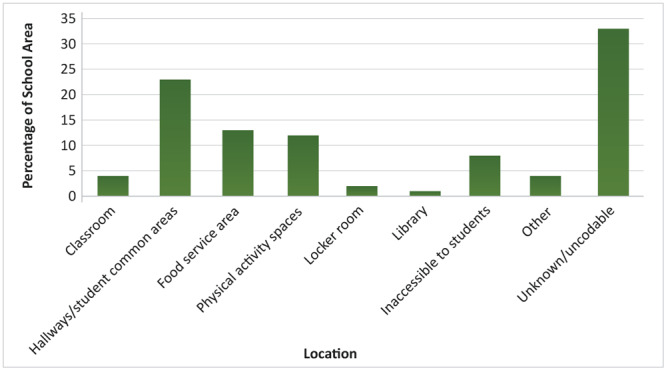
School locations where drinking water was sampled for lead testing, by percentage of school area served.

**Table:** 

Location	Percentage of School Area
Classrooms	4
Hallways/student common areas	23
Food service area	13
Physical activity spaces	12
Locker room	2
Library	1
Inaccessible to students	8
Other	4
Unknown/uncodable	33

## Discussion

Our study is one of the first in-depth examinations of a US state’s drinking water quality and testing in public schools (11) and is the first study to look at overall water quality in schools that includes both data on compliance with water system health standards and school lead-testing program uptake and results. We found that schools serving a high proportion of students from racial/ethnic minority backgrounds were more likely to receive water from water systems with a history of drinking water standard violations than schools serving few racially/ethnically diverse students. Suburban schools were also less likely to receive water from noncompliant water systems than schools in cities. Regarding lead testing, rural and town schools were less likely to test drinking water for lead than city schools. Although few schools were in violation of California state standards for lead, violations increased when the FDA standard was applied. Most schools with lead exceedance issues (violations) received water from compliant water utility sources. Although most water utilities were compliant, lead can enter the water through lead-bearing pipes, solder, or fixtures after leaving the water source (20).

AB 746 was passed to protect students from the harm of lead-contaminated water by testing drinking water sources and ensuring that potable water is provided if the source exceeds the action level for lead. Our analysis showed that 73% of schools in our sample completed testing by the time of our study. Despite fair uptake of the testing program, some challenges occurred in implementation. Barriers to implementation included testing water sources that may have been inaccessible to children and failure to label the locations of water source samples.

Historically, people from low-income and racial/ethnic minority groups have faced barriers in accessing safe drinking water (21,22). In our study, we found that schools serving a high proportion of students from racial/ethnic minority backgrounds were more likely to receive water from systems with a history of water quality violations. This finding adds to mounting evidence of the need for additional resources to help low-income, racial/ethnic minority populations address inequities in drinking water quality (23,24).

The geographic setting of schools was an important predictor of participation in the lead-testing program. Rural schools were less likely to test for lead in drinking water than schools in other geographic areas. This finding aligns with existing literature that suggests that rural schools may have decreased ability to actively engage in noncritical actions, such as drinking water testing, because of lack of financial resources and staff capacity (25). Although water utilities are mandated to assist with testing through the state program, schools would still need to initiate and manage the testing process and remediation efforts, which may be more challenging for under-resourced schools to oversee and fund.

The lead action level that a jurisdiction sets could have significant health and financial implications for schools participating in school-based drinking water testing and remediation programs. In our sample of California schools, roughly 3% of schools that tested had at least 1 sample that exceeded 15 ppb, the current federal and California state action level. Our findings are consistent with the findings from the early adopters study of 2,403 California state schools conducted by Cradock et al (8). The Cradock study also highlighted wide variation in the proportion of schools with a sample that exceeded a state’s chosen action level, ranging from 2% in Alabama (20 ppb threshold) to 78% in Washington, DC (1 ppb threshold) (8). Although the Environmental Protection Agency sets their action level at 15 ppb, any amount of lead in water has potential health consequences (26). In our study, had California’s action level been lowered to 5 ppb, a 9-fold increase in the proportion of schools required to take steps to remediate their drinking water would have resulted. Although the main lead mitigation strategies all have various cost implications (20), an increase in the number of schools facing lead contamination in drinking water would increase the remediation cost for schools and districts. Because California’s state testing measured lead levels only as low as 5 ppb, potential implications for even lower lead action levels remain unknown. States should test to nondetectable levels in water safety programs to capture the data necessary to inform and appraise progressive water safety policies.

AB 746 was instituted without clear implementation and reporting guidelines. Instead, water utility services were encouraged to work with schools to devise sampling plans for each school (27). As in other statewide examinations, we found a wide range in implementation of the law. For example, although some schools tested only 1 tap, others tested as many as 76. Schools that test fewer taps may be less likely to adequately capture the risk of elevated lead in drinking water than schools that test a greater number of taps. Inconsistency in testing practices hinders states or the federal government from accurately identifying which schools or regions require additional support for lead remediation. Additionally, 51% of schools in our study sent in at least 1 water sample from an unknown or unidentifiable location, and the locations of one-third of all sites tested were unidentifiable. Unclear tap identification makes it difficult to determine whether students are drinking from these taps and, consequently, whether the results accurately assess the risk of children consuming unsafe drinking water. We also found variability in the types of water sources and locations from which drinking water was sampled. Sixty-one percent of schools did not test a water source in a food service area, and 29% of schools tested locations that were not easily accessible to children. Given that federal and state law requires that potable water be made available in food service areas, state school water testing programs should require that at least 1 water source in food service areas be tested for lead. Because lead has the greatest effect on the development of young children, policy makers and schools should prioritize sampling potable drinking water sources accessible to children.

Findings from our postimplementation study also have important policy implications for California AB 2370, which requires all licensed childcare facilities to test taps for lead by 2021 and every 5 years after initial testing (28). Findings from our study suggest that AB 2370 should be accompanied by detailed protocols that provide clear guidelines on the number of taps facilities should test, the required location of taps for testing, and clear naming conventions and reporting procedures. Evaluations of lead-testing programs may also play a role in informing national water safety regulations (8). The EPA’s proposed Lead and Copper Rule would mandate that public water systems offer to test for lead in the drinking water in 5 taps at each school and 2 taps at each licensed childcare facility. Findings from our study could be used to inform how federal oversight of school and childcare water safety practices is implemented, managed, and evaluated to ensure that effective, evidence-based, and comprehensive policies are carried out with maximum effectiveness.

A key strength of this study is that it is the first comprehensive examination of water quality practices, including lead testing, in schools. The collation of school water safety information with water utility compliance data from several disparate state and independent sources provides a comprehensive study of water safety in California public schools, which are often analyzed separately, without comparison across schools. It is also one of the first to provide an in-depth description of the implementation of a state school lead-testing program following the testing and reporting deadlines (11).

Despite these strengths, our study has some limitations. Our sample included only 240 schools (2.2% of eligible schools in California). Although the sample size limited our power to examine school and student factors related to water safety testing practices and results, the demographics of study schools appeared similar to California schools in terms of the proportion of low-income and racial/ethnic minority students. Additionally, our methodology grouped water system compliance into those schools with a history of compliance and those with a history of noncompliance. This grouping may have limited the ability to detect nuances and differences between water systems that were currently noncompliant and systems that corrected issues promptly and returned to compliance. Because the state testing program measured lead contamination only down to 5 ppb, we were unable to understand the implications of more stringent testing standards, such as the 1 ppb threshold set by the American Academy of Pediatrics (29). Schools that provide their own water systems, such as those supplied by wells, were exempt from this program because they are required to regularly test for lead under the Safe Drinking Water Act (30). Most schools were served by a water utility; only about 500 schools in California operate as their own water suppliers (27). Rural schools are more likely to use wells than other schools, thus testing frequency in rural schools may be underestimated in our study, which only investigated testing pursuant to AB 746. Lastly, because our study focused on California schools, results may not be generalizable to other states. Nevertheless, study findings provide important considerations for other states as they seek to institute or improve their state testing programs.

Access to safe drinking water in schools is essential to help avoid the developmental and health consequences for children associated with consuming contaminated water, dehydration, or excessive intake of sugary beverages. Federal and state drinking water regulations and state testing programs for lead in schools seek to promote equitable access to safe drinking water in all schools. Despite these efforts, unclear regulation and ambiguous guidelines result in variable implementation in the type, number, and location of water sources tested across schools, which may further exacerbate existing racial/ethnic and income-related disparities in safe drinking water access. Moving forward, clear and detailed implementation protocols should accompany regulations to improve equitable implementation of safe drinking water policies. As states, localities, and schools set new action levels for lead in drinking water, they should review the benefits of lowering action levels to minimize the health harms of lead exposure in the context of the financial implications of such standards for schools, districts, and states, which currently bear most of the costs.
